# A systematic review of effectiveness and safety of different regimens of levonorgestrel oral tablets for emergency contraception

**DOI:** 10.1186/1472-6874-14-54

**Published:** 2014-04-04

**Authors:** Mohammad Shohel, Mohammad Mahfuzur Rahman, Asif Zaman, Mir Muhammad Nasir Uddin, Md Mamun Al-Amin, Hasan Mahmud Reza

**Affiliations:** 1 Department of Pharmaceutical Sciences, North South University, Dhaka 1229, Bangladesh; 2 Department of Pharmacy, University of Chittagong, Chittagong, Bangladesh

**Keywords:** Levonorgestrel, Oral contraceptive, Emergency contraception, Two dose regimen, Postcoital contraception, Single dose strategy, Contraceptive efficacy, Unintended pregnancy, Postcoital hormonal contraceptives

## Abstract

**Background:**

Unintended pregnancy is a complex phenomenon which raise to take an emergency decision. Low contraceptive prevalence and high user failure rates are the leading causes of this unexpected situation. High user failure rates suggest the vital role of emergency contraception to prevent unplanned pregnancy. Levonorgestrel - a commonly used progestin for emergency contraception. However, little is known about its pharmacokinetics and optimal dose for use. Hence, there is a need to conduct a systematic review of the available evidences.

**Methods:**

Randomized, double-blind trials were sought, evaluating healthy women with regular menstrual cycles, who requested emergency contraception within 72 h of unprotected coitus, to one of three regimens: 1.5 mg single dose levonorgestrel, two doses of 0.75 mg levonorgestrel given 12 h apart or two doses of 0.75 mg levonorgestrel given 24 h apart. The primary outcome was unintended pregnancy; other outcomes were side-effects and timing of next menstruation.

**Results:**

Every trial under consideration successfully established the contraceptive effectiveness of levonorgestrel for preventing unintended pregnancy. Moreover, a single dose of levonorgestrel 1.5 mg for emergency contraception supports its safety and efficacy profile. If two doses of levonorgestrel 0.75 mg are intended for administration, the second dose can positively be taken 12–24 h after the first dose without compromising its contraceptive efficacy. The main side effect was frequent menstrual irregularities. No serious adverse events were reported.

**Conclusions:**

The review shows that, emergency contraceptive regimen of single-dose levonorgestrel is not inferior in efficacy to the two-dose regimen. All the regimens studied were very efficacious for emergency contraception and prevented a high proportion of pregnancies if taken within 72 h of unprotected coitus. Single levonorgestrel dose (1.5 mg) can substitute two 0.75 mg doses 12 or 24 h apart. With either regimen, the earlier the treatment is given, the more effective it seems to be.

## Background

Access to emergency contraception and its need for increasing awareness and convenience is very important nowadays. Post-coital or emergency contraception is a simple, reliable and effective strategy. However, the complications associated with the emergency contraception are needed to be addressed before its full potential is realized. The most challenging aspect is that healthcare personnel and potential users are both quite ignorant of this approach [1]. The other major obstacle which prevents the extensive use of emergency contraception is that both potential users and doctors believe that such approaches to contraception are accompanied with frequent and severe side-effects [2-4]. Previous data suggest that, the significance of emergency contraceptive pills cannot be ignored any longer, since one million abortions and two million unwanted pregnancies could be prevented in the U.S. each year if its widespread use could be ensured [5].

Currently available commonly acknowledged hormonal method of post-coital contraception was first described by Yuzpe and Lancee [6] and Yuzpe *et al.*[7]. Based on the findings of these authors, each dosage included the combination of 200 μg ethinyl oestradiol and 2 mg dl-norgestrel given in two divided doses where the second dose was given after 12 h of the first dose, within 72 h of unprotected intercourse. Though the pregnancy rate was only 1.6%, there was a relatively high prevalence of side-effects (~50% of cases). Side effects including nausea and/or vomiting, this leads to decreased compliance of patients [8]. Even though the Yuzpe regimen has become an established and recommended method of emergency contraception, there is a need to reduce the incidence of adverse effects while maintaining effectiveness [9].

Levonorgestrel (LNG) is a synthetic, biologically active progestogen, structurally related to 19-nortestosterone, which may be used alone or in combination with estrogens for the prevention of unintended pregnancies following unprotected coitus [10]. LNG is only sought as a supportive method for irregular rather than regular use and LNG is intended to be used immediately after intercourse but prior to pregnancy has become recognized [11]. LNG is also safe and effective in women with type I and type II diabetes [12]. Levonorgestrel-only emergency contraceptive has an efficiency rate of 89% if it is used correctly within 72 h after unprotected intercourse. LNG also diminishes the risk of pregnancy, following a single act of mid-cycle unprotected sexual intercourse, from 8% on average, to 1.1% [13]. Every time the dose is delayed 12 h after starting emergency contraception treatment it reduces efficacy by about 50% [14].

LNG works in several different mechanisms depending on the cycle day of unprotected intercourse and the day on which the treatment is started. LNG may inhibit the process of ovulation, fertilization or implantation [15-20]. However, there is no direct clinical indication that supports these mechanisms. Emergency contraception is effective only before a fertilized egg is implanted because it can’t disrupt an existing pregnancy, i.e., emergency contraceptives are not abortifacient [21]. Several studies suggested that the lowest concentration required to prevent ovulation is 0.48 nmol/L [22]. Hence, appropriate dosing is critical to ensure the prevention of pregnancy.

For emergency contraception, there is pharmacokinetic evidence for two doses of 0.75 mg of levonorgestrel, 12 h apart [23-25]. The oral bioavailability of levonorgestrel is > 90%. However, this has been reported to depend on the dosage form and to be affected by a concomitant administration of estrogens [26,27]. The peak concentration after a single oral dose of 0.75 mg of levonorgestrel is attained within 1 h after administration, with a half-life that is within 20 to 60 h [23-25]. However, not much is known about the pharmacokinetics of a single dose of 1.5 mg of levonorgestrel in fertile women during the periovulatory period of the menstrual cycle [23].

The purpose of this study was to compare the efficacy and side-effects of two treatments, when administered up to 72 h after unprotected coitus: a single dose of 1.5 mg levonorgestrel; and two separate doses of 0.75 mg levonorgestrel that are given 12 and 24 h apart. The main outcomes were pregnancy rates, proportions of pregnancies prevented, side-effects and timing of the first menstrual period after treatment. We also thought to analyze the effect of treatment delay on the efficacy of contraception.

Our idea was that all the regimens in consideration would have similar efficacy in preventing pregnancy. We also analyzed how the timing of contraception in relation to coitus influenced treatment effectiveness.

## Methods

### Criteria for inclusion and exclusion

Randomized, double blind studies were selected for this review. The literature which included women of fertile age, compared one and/or two doses of levonorgestrel taken in different regimens and described the kinetics or dynamics of the drug were also reviewed. Reasons for exclusion including non-English studies, literatures involving non-human models, studies which compared levonorgestrel with other emergency hormonal contraceptives and publications concerned with dosage forms other than oral tablets.

### Data sources

MEDLINE, HighWire Press, Elsevier, PubMed, Google Scholar, Springerlink, EBSCO, and Wolters Kluwer Health databases were searched from 1967–2012 using the key terms emergency contraception, postcoital contraception, postcoital contraceptives, levonorgestrel, single dose and double dose. The trials identified with our search strategy were initially checked for duplicates and relevance for the review by looking at the titles and abstracts. If it was not possible to exclude a publication by looking at the title or the abstract, the full paper was retrieved. Decisions on which trials to include were independently made by two reviewers. Bibliographies of retrieved articles for additional studies were also searched. We limited the search to English-language reports involving humans. Studies were selected where patient outcomes or pharmacokinetics were assessed with alternative levonorgestrel-only emergency contraception (EC).

### Data extraction

**Figure 1:**
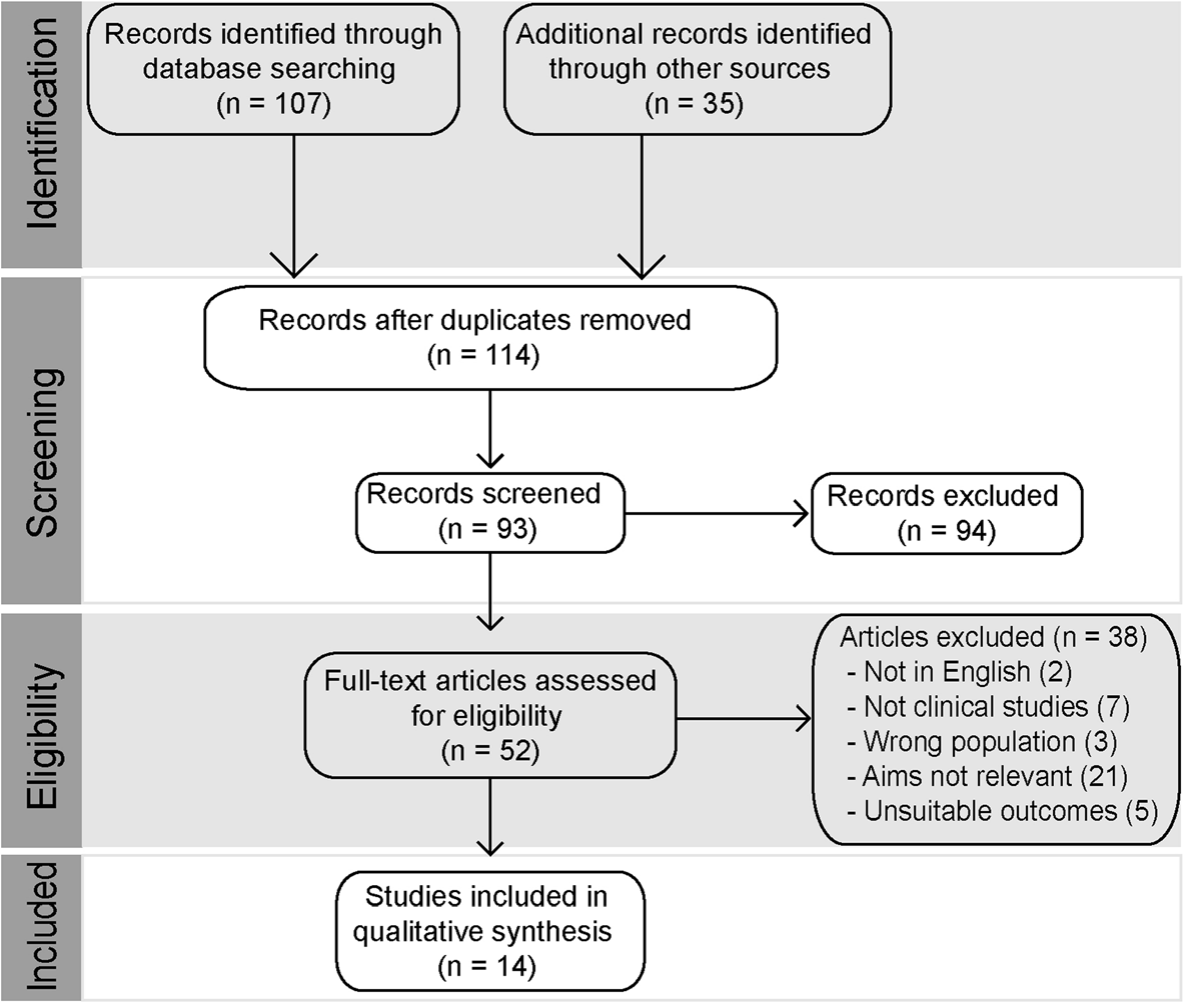
Study selection flow diagram.

Two authors independently reviewed the search results for reports of studies of any design of hormonal drugs taken orally for contraception, immediately before or after each act of intercourse during one or more menstrual cycles. We found 114 relevant studies (Figure 1). We excluded eighty one trials from our review because they contained incomplete information on the drug regimen, number of pregnancies, or the duration of data collection and four because only a partial report or abstract was available. Of the remaining 29 studies, 14 provided data on regimens containing levonorgestrel. The others described studies comparing with other compounds and therefore those ten studies were omitted from this review. Discrepancies were resolved by discussion and consultation with other reviewers including clinicians if needed.

### Outcome measures

Outcome measures included pregnancy rates, percent of pregnancies prevented and side-effects. From kinetic viewpoint, the area under the plasma concentration versus time curve to last time, area under the plasma concentration curve to infinity, peak plasma concentration, time of peak plasma concentration, elimination rate constant and terminal half-life were observed. We also assessed the outcome by comparing the length of the cycle, bleeding patterns, follicular rupture and ovulation period, ovulatory dysfunction, length of luteal phase, day of maximal luteal serum progesterone (P4), follicular outcome, luteal serum glycodelin and the effect on total lutenizing hormone (LH).

Finally, we used the Preferred Reporting Items for Systematic reviews and Meta-Analyses (PRISMA) criteria for reporting systematic reviews of studies that evaluate healthcare interventions, a quality appraisal tool.

## Results

Six of the 14 trials were randomized, double blind studies of a single regimen. Eight trials included multiple groups of women given levonorgestrel tablets of different doses. The method of treatment allocation was not described, so we considered each group as a separate case series. The total number of treatment groups analyzed across all studies was 28. Of these groups, 11 evaluated tablets containing 0.75 mg levonorgestrel, and the others evaluated tablets containing other doses (Table 1).

**Table 1:** Study design and demographic data

**Study (year) [ref]**	**Country**	**Population characteristics**		**Study design**		
		**No. of subjects**	**Age (years)**	**Type of trial**	**Dose**	**Dose frequency**
Arowojolu *et al.*, (2002) [30]	Nigeria	1160	19-35	Prospective comparative study	0.75 mg & 1.5 mg	Once & two split doses 12 h apart
Croxatto *et al.*, (2004) [33]	Chile, Dominican Republic	58	18-40	Two-center, randomized, double-blind, placebo-controlled study	0.75 mg	Two doses given 12 h apart, or a single dose
Dada *et al.*, (2010) [31]	Nigeria	3022	20-33	Randomized, controlled, double-blind, multicentre, non-inferiority trial	0.75 mg & 1.5 mg	Two doses, 12 h apart vs. a single dose
Durand *et al.*, (2001) [18]	Mexico	45	29-35	Randomized, comparative study	0.75 mg	Two doses taken 12 h apart, at 0 h & after 48 h
Durand *et al.*, (2005) [34]	Mexico	30	29-35	Randomized, comparative study	0.75 mg	Two doses taken 12 h apart, at 0 h & after 48 h
Gainer *et al.*, (2007) [28]	Chile	12	18-32	—	1.5 mg	Once
Hapangama *et al.*, (2001) [19]	Scotland	12	26-41	Prospective, randomized, double-blind, crossover study	0.75 mg	Two doses given 12 h apart
Johansson *et al.*, (2002) [23]	Dominican Republic	5	18-45	Randomized, comparative study	0.75 mg & 1.5 mg	Once, two doses given 12 h apart, two doses given 24 h apart
Kook *et al.*, (2002) [24]	USA	16	19-44	Open label study	0.75 mg	Twice, taken 12 h apart
Ngai *et al.*, (2005) [32]	China	2071	20-34	Double-blind, randomized trial	0.75 mg	Every 24 h & every 12 h
Noé *et al.*, (2010) [35]	Chile	388	18-38	Prospective, open-label, single-drug, single dose, uncontrolled clinical trial	1.5 mg	Once
Raymond *et al.*, (2006) [36]	USA	120	18-38	Case-series study	1.5 mg	Once
Sambol *et al.*, (2006) [29]	USA	22	13-16	Prospective, single-period, single center, open-label clinical trial	0.75 mg	Once
Tremblay *et al.*, (2001) [25]	South Africa	24	18-26	Open, observer-blind, randomized study	0.75 mg	Once, two doses given 12 h apart, two doses given 24 h apart

Levonorgestrel has a prolonged half-life of around 24.4 h. This rationalizes considering single-dose LNG emergency contraception as well as two doses taken 12–24 h apart. Pharmacokinetics of levonorgestrel was evaluated in a study using different dosing strategies in young, healthy, women [25]. This trial involved 24 women who were randomized to three levonorgestrel treatment groups: a single 0.75 mg dose, two 0.75 mg doses taken 12 h apart and two 0.75 mg doses taken 24 h apart. The pharmacokinetic profiles were similar with all three treatments. Plasma concentrations of levonorgestrel were 9.6 ± 0.38 nmol/L at 12 h and 6.2 ± 0.53 nmol/L at 24 h after the first 0.75 mg dose. Effective concentrations greater than 0.48 nmol/L were maintained for 48–60 h after the 12 and 24 h doses. The statistical investigations indicated no significant differences between groups in any of the pharmacokinetic parameters i.e. peak plasma concentration (C_max_), time to reach peak plasma concentration (t_max_), elimination rate constant (K_el_), half-life (t_1/2_) and area under plasma concentration-time curve AUC.

An investigation on the pharmacokinetics of 1.5 mg levonorgestrel in lactating women was also evaluated [28]. Twelve healthy breastfeeding participants received 1.5 mg of LNG. For 72 h after dosing, the women abstained from nursing and fed their infants with milk which was frozen previously. Sequential milk and blood samples were collected and assessed for LNG and sex hormone binding globulin for 120 h. LNG concentrations peaked in milk and plasma in 2-4 h and 1-4 h after dosing, respectively. Concentrations in milk were similar to those in plasma but were consistently lower. Projected infant exposure to LNG is 1.6 mg on the day of dosing, 0.3 mg on the second day and 0.2 mg on the third day.

Another study examined plasma LNG concentrations and pharmacokinetics after oral administration of a single 0.75 mg tablet [24]. Blood samples were collected sequentially over 72 h after dosing in a fasting state. C_max_ of LNG was 14.1 ± 7.9 ng/mL and t_max_ was 1.63 ± 0.74 h. Plasma concentration of LNG vs. time profiles were subjected to noncompartmental pharmacokinetic analysis for determining half-lives, apparent oral clearances (Cl/F), apparent volumes of distribution after oral administration (V/F) and mean residence time (MRT). The half-lives ranged from 16.2 h to 32.3 h (mean = 24.4 ± 5.3 h). The Cl/F was 7.06 ± 2.69 L/h, V/F was 260 ± 129 L and MRT was 27.8 ± 5.2 h.

In a different study, the pharmacokinetics of levonorgestrel between female adolescents and adults were compared [29]. A total of 259 levonorgestrel plasma concentrations in 22 female adolescents were investigated. On average, C_max_ was 6.72 ng/mL and occurred at 1.43 h after dosing. With a mean 9.5% contribution from extrapolation, average AUC_0–∞_ was 86.1 ng h/mL. Mean CL/F was 145 ml/min and mean V/F was 267 L (Table 2). However, the data from an adult study (304 levonorgestrel plasma concentrations in 16 women) were also reconsidered. Adolescents had a mean levonorgestrel C_max_ that was almost half of that detected in adults, with a mean ratio of 0.53 and a 90% CI (Table 2). The difference among the groups with respect to AUC_0–∞_ was of borderline significance, with a mean ratio of 0.77. Also, the variance between the treatment groups with respect to CL/F was marginally significant, with a mean ratio of 1.31.

**Table 2:** Pharmacokinetic comparison of different dosage regimens of levonorgestrel

**Study (year)**	**Dose**	**P**_**k **_**parameters**								**Side effects**
		**AUC**_**0–12 **_**(ng-h/ml)**	**AUC**_**0–24 **_**(ng-h/ml)**	**AUC**_**0-t **_**(ng-h/ml)**	**AUC**_**0-∞ **_**(ng-h/ml)**	**C**_**max **_**(ng/ml)**	**t**_**max **_**(h)**	**K**_**el **_**(1/h)**	**t**_**1/2 **_**(h)**	
Gainer *et al.*, (2007) [28]	1.5 mg	28.6	45.0	252.8	262.6	15.4	2.0	----	29.3	----
Johansson *et al.*, (2002) [23]	0.75 mg & 1.5 mg	A:49.9	A:81.3	A:139.5	----	A:7.9	A:1.8	----	A:43.7	Nausea, sleepiness, headache.
		B:51.9	B:73.2	B:136.0		B:8.4	B:1.4		B:32.0	
		C:88.8	C:130.8	C:290.9		C:12.4	C:2.5		C:43.3	
Kook *et al.*, (2002) [24]	0.75 mg	----	----	111.8	123.1	14.1	1.63	0.030	24.4	Menstrual irregularity
Sambol *et al.*, (2006) [29]	0.75 mg	----	----	----	86.1	6.72	1.43	----	21.2	Headaches, dizziness, nausea, cramps
Tremblay *et al.*, (2001) [25]	0.75 mg	----	----	----	133.02	----	2.27	0.042	21.72	----

AUC_0-t_: Area under the plasma concentration time curve calculated by the trapezoidal rule from time 0 until the last time point.

AUC_0-∞_: Area under the plasma concentration time curve corrected for the previous administration and extrapolated to infinity.

A: One tablet of 0.75 mg levonorgestrel at time −12 h and one tablet at time 0.

B: One tablet of 0.75 mg levonorgestrel at time 0.

C: One tablet of 0.75 mg levonorgestrel at time −24 h and one tablet at time 0.

In addition, a group of investigators studied the pharmacokinetics of levonorgestrel where compared dosages were two 0.75 mg doses taken 12 h apart, two 0.75 mg doses taken 24 h apart, and a single 1.5 mg dose [23]. In this trial, five women were randomized to be given all three treatments. They also received a four week washout period between the treatments. t_max_ was estimated 1.5–1.8 h after each of the two 0.75 mg doses and 2.6 h after the single 1.5 mg dose. C_max_ after the 1.5 mg single dose was about 50% greater than those after each 0.75 mg dose. The second dose of both the 12 and 24 h double dose regimens revealed a slightly higher C_max_ than that of the first dose. All three groups had similar mean LNG concentrations at 48 h.

For finding an acceptable levonorgestrel regimen for emergency contraception, a two-dose regimen 0.75 mg levonorgestrel 12 h apart and a single dose 1.5 mg levonorgestrel were studied in 1118 Nigerian women [30]. Eleven pregnancies were reported, among them 7 were in the two-dose group and 4 in the single dose group. The relative risk of pregnancies was similar in both groups. Also, the estimated success rate of 86.80% in the 0.75 mg group was significantly lower than the 92.99% for the 1.5 mg group (Table 3). Minor side effects were reported such as nausea, vomiting, menorrhagia, lower abdominal pains, headache, breast tenderness and dizziness. Considerably more women who were in the high-dose group reported headache, breast tenderness and heavy menstrual flow.

**Table 3:** Clinical statistics of levonorgestrel

**Outcome measures**	**Dosage regimen**		
	**1.5 mg**	**0.75 mg (12 hourly)**	**0.75 mg (24 hourly)**
Pregnancy rate (%)	0.64^(a)^ – 1.28^(b)^	0.57^(b)^ – 2.0^(c)^	1.9^(c)^
Prevented fraction (%)	92.99^(a)^ – 94.7^(b)^	75.0^(c)^ – 95.2^(b)^	72.0^(c)^
Side effects (more prominent)	Headache, breast tenderness, heavy menses, fatigue, vomiting	Nausea, diarrhea, lower abdominal pain, delay of menses	Dizziness

^(a)^[30]; ^(b)^[31]; ^(c)^[32].

Another study considering the same dosage regimen among 3022 Nigerian women revealed similar effectiveness between the treatment groups. In the two-dose regimen, post-treatment pregnancy proportions were 0.57% whereas in the single-dose regimen, the proportion was 0.64% (Table 3). Most of the women menstruated in the first day of expected menses and the groups did not diverge regarding reported side effects. In general, the most common adverse event was nausea, which was accounted for about 22% of participants in both groups. Some of the participants also experienced fatigue, dizziness, headache, breast tenderness and lower abdominal pain [31].

A different study compared the efficacy of two doses of 0.75 mg levonorgestrel among 2071 Chinese women, given at either a 12 or 24 h interval, with the treatment period extended to 120 h after unprotected coitus [32]. The crude pregnancy rate was 2.0% in the 12 h group and 1.9% in the 24 h group. The prevented fraction of pregnancies was considered to be 75% in the 12 h group and 72% in the 24 h group (Table 3). The effectiveness of the 12 h regimen dropped significantly when there were further acts of coitus after treatment which was not observed in the 24 h group. The overall side-effects were mild and tolerable. Nausea and vomiting occurred in 10% of women whereas diarrhea and breast tenderness was significantly more common in the 12 h group. Also, the timing of menses did not vary between the two regimens.

A randomized, double-blind, placebo-controlled study evaluated to what degree the usual dose of levonorgestrel or a single dose, given in the follicular phase, affects the ovulatory process during the subsequent period of five days [33]. Time of treatment was determined by the diameter of the dominant follicle. Every woman had equivalent diameter allocated for all her treatments. Follicular rupture failed to take place during the five day period in 44%, 50% and 36% of cycles respectively with the standard, single dose and placebo. Ovulatory dysfunction occurred in 35%, 36% and 5% of standard, single dose or placebo cycles, respectively, which is characterized by follicular rupture associated with absent, blunted or untimely gonadotropin surge.

Durand M et al. [18] studied the effects of short-term administration of levonorgestrel at different phases of the ovarian cycle on the pituitary-ovarian axis, corpus luteum function and endometrium. The participants were studied during two menstrual cycles. Transvaginal ultrasound and serum LH were performed in both cycles from the detection of urinary LH until ovulation. During the complete luteal phase, serum estradiol and progesterone were measured. An endometrial biopsy was also taken, which revealed the endometrial histology as normal in all ovulatory-treated cycles. Finally, they suggested that “intervention of LNG with the mechanisms initiating the LH preovulatory surge depends on the stage of follicle growth”. Thus, anovulation caused from disrupting the regular development and/or the hormonal activity of the developing follicle, only when LNG was given in the preovulatory phase.

Durand M et al. [34] analyzed endometrial expression and serum glycodelin concentrations during the luteal phase following oral levonorgestrel at various stages of the ovarian cycle. All the participants were studied during two consecutive cycles, namely a control cycle and the treatment cycle. During the luteal phase, serum progesterone and glycodelin were measured daily and an endometrial biopsy was taken for immuno-histochemical glycodelin-A staining. Levonorgestrel altered the luteal phase secretory pattern of glycodelin in serum and endometrium. The results accounted for the action of LNG in EC in those women who take LNG before the LH surge, based on the potent gamete adhesion inhibitory activity of glycodelin-A.

In a different study, 0.75 mg LNG was administered twice right before ovulation, to test whether LNG acts as an emergency contraceptive by stopping the pre-ovulatory LH surge and thereby delaying ovulation [19]. LNG was taken by twelve women on or before the day of the first significant increase in urinary LH in 12 cycles. The LH peak and the onset of next menses were delayed (16.8 days) in 30% participants. Despite a normal LH peak and cycle length, one woman did not ovulate at all. LNG did not affect ovulation or the cycle length in the remaining eight participants, but the length of the luteal phase and the total luteal phase LH concentrations were considerably reduced.

Noé G et al. [35] aimed to evaluate whether levonorgestrel administered after ovulation is equally effective to LNG administered before ovulation. Blood samples were taken for analysis of LH, estradiol and progesterone concentrations on the day of LNG administration and during five days’ follow-up. Vaginal ultrasound analyses were also done for size of the leading follicle and/or corpus luteum. 122 women among the total of 388 women had coitus on fertile cycle days according to ultrasound and endocrine findings. 87 women were in days −5 to −1 and 35 women were in day 0 or beyond at the time of LNG intake. Estimated numbers of pregnancies among the 87 and 35 women were 13 and 7 respectively, while actually 0 and 6 pregnancies respectively occurred.

Menstrual bleeding pattern after using 1.5 mg levonorgestrel in a single dose was also examined [36]. The observed bleeding patterns after treatment were compared with usual patterns reported by the participants and with patterns observed in a previous study on women who had not taken any emergency contraceptives. Treatment in the first three weeks of the menstrual cycle significantly reduced that cycle as compared with both the usual cycle length and with the cycle duration in a comparison group. The magnitude of this outcome was greater if the pills were taken earlier. Additionally, the duration of the first menstrual period after treatment increased significantly with cycle week of treatment. This timeline was longer in participants who used the treatment than in those who did not.

## Discussion

From the pharmacokinetic perspective, levonorgestrel was rapidly absorbed after either one single or two administrations at a 12 or 24 h time interval of levonorgestrel 750 μg oral tablets. Also, the ADME profiles of levonorgestrel following the three different treatment regimens were similar [25]. The blood levels following administration of the first of two tablets (at 12 h interval) in the emergency contraceptive regimen were maintained for a sufficiently long period of time to prevent further unwanted fertilization [24]. Nevertheless, levonorgestrel was generally well tolerated by adolescents (single dose, 0.75 mg). The differences in observed total concentration were not likely to be seen in the unbound concentration. So, there is no reason based on pharmacokinetic data to expect that adolescents will experience greater adverse effects or lesser therapeutic effects after administration of the usual dose of LNG EC [29].

The AUC after administration of one single dose of 1.5 mg LNG was greater than two doses of 0.75 mg with a 12 or 24 h interval. Therefore, the administration of a single larger dose was more effective than the same amount of LNG divided into two doses [23]. On the other hand, nursing mothers might need EC but when a medication is taken by nursing women, the risks and benefits of taking that drug must first be weighed. Since LNG passes rapidly into the milk (single dose, 1.5 mg), the period of maximum LNG excretion in milk should be avoided and nursing should be discontinued and milk discarded for an interval of at least 8 h, but not longer than 24 h, after the use of EC [28].

From clinical point of view, the single 1.5 mg regimen of levonorgestrel appeared more effective than the split doses of 0.75 mg taken twice 12 h apart, not in terms of raw pregnancy rate or relative risk but in terms of effectiveness rate. The earlier each regimen was given after unprotected sexual intercourse, the more was the efficacy [30]. However, some authors claimed that the regimen of single-dose levonorgestrel was non-inferior and as well tolerated as the two-dose regimen. The patients requested EC did so because they were not using contraception at coitus. So it was suggested that levonorgestrel EC should be offered up to 5 days after unprotected intercourse [31]. In case of a 0.75 mg regimen, the 24 h double dose levonorgestrel regimen was as effective as the 12 h regimen for emergency contraception up to 120 h after unprotected intercourse [32]. All the treatment regimens were well tolerated except some common side-effects and did not have any lasting effect on the individuals.

While assessing the mode of action of levonorgestrel, it was observed that LNG used for EC prevents pregnancy primarily by interfering with the ovulatory process and method failures are most likely due to treatment given too late to effect such intervention. No association was found between the usage of LNG and the risk of pregnancy complications, major congenital malformations or any other adverse pregnancy outcomes in case of its failure as an emergency contraceptive [37,38]. In addition, single administration of 0.75 mg LNG was at least as effective as the standard two-dose regimen for inhibiting ovulation [33]. However, preovulatory administration of LNG suppresses ovulation in most but not all cases. Hinderance of LNG with the mechanisms involved in initiating the Luteinizing hormone preovulatory surge depends on the stage of follicular development. Thus, anovulation results from disrupting both normal maturation and hormonal natural action of a growing follicle [18]. Other plausible mechanism of actions of LNG such as retardation of the endometrium, interfering with sperm motility and changing cervical mucus [19]. This is because the in vitro effect of LNG as EC on sperm fertilizing capacity and embryo development remains poorly understood in various other potential studies [39-41].

Hormonal EC with LNG can alter endometrial glycodelin secretion in two important phases of the cycle when taken before the LH surge [42]. The first is during the fertile window and the second is the phase of uterine receptivity, both of which are of interest because of their antifertility activity [34]. However, it was observed that neither the glycodein level nor the proportion of motile sperm and cervical mucus is influenced by LNG [43]. Also, emergency contraception of levonorgestrel (1.5 mg) in a single dose taken in the first 3 weeks of the menstrual cycle shortened that cycle. The degree of this effect was higher than the earlier the pills were taken. This regimen taken later in the cycle had no effect on cycle length but caused prolongation of the next menstrual episode [36]. So it is important to note that, LNG-EC is very effective in preventing pregnancy when it is administered before ovulation, but it is ineffective in such once fertilization has occurred [44]. That’s why LNG-EC is less effective than regular contraceptives and its use should be controlled to emergency situations [35].

### Limitations

The quality of several reports was poor. A number of reports lacked details about the study procedures, including the inclusion criteria, frequency of follow-up contacts, or method of pregnancy ascertainment. Seven reports did not specify the intended duration of follow-up, although five of these did present detailed information about continuation, discontinuation, and loss to follow-up in each month or 3 month period. Some studies did not mention the proportion of participants who may have continued to use the method after the last follow-up visit and who therefore may have had pregnancies that occurred during method use but that were not ascertained by the researchers. Studies were not able to explore the possibility that prevention of pregnancy may occur by some other natural means rather than effect of drug. We could not determine whether these shortcomings were the result of poor study quality or simply inadequate reporting. However, several studies that provided more than two thirds of the data in this review were well designed and clearly reported.

## Conclusions

For emergency contraception, the US-FDA approves two doses of levonorgestrel 0.75 mg. The first dose should be taken within 72 h of unprotected intercourse and the second dosage, 12 h after the initial dosage. On the other hand, a single dose of levonorgestrel 1.5 mg is also safe and effective for emergency contraception. Based on previous research findings, pharmacokinetic data and patient outcomes; the single dose strategy is warranted. Additionally, in case of two doses of levonorgestrel 0.75 mg, the second dosage can be taken within 24 h after the first dosage and the effectiveness of the drug would not be compromised. The clinical significance of this information is critical for clinicians instructing patients or prescribing levonorgestrel for emergency contraception.

## Abbreviations

LNG: Levonorgestrel; LH: Lutenizing Hormone; P4: Day of maximal luteal serum progesterone; Cmax: Peak plasma concentration; tmax: Time to reach peak plasma concentration; Kel: Elimination rate constant; t1/2: Half-life; AUC: Area under plasma concentration-time curve; AUC0-t: Area under the plasma concentration time curve calculated by the trapezoidal rule from time 0 until the last time point; AUC0-∞: Area under the plasma concentration time curve corrected for the previous administration and extrapolated to infinity; Cl/F: Apparent oral clearances; V/F: Apparent volumes of distribution after oral administration; MRT: Mean residence time; EC: Emergency contraception/contraceptive; ADME: Absorption, distribution, metabolism, excretion; FDA: Food and drug administration.

## Competing interests

The authors have no competing financial interests in relation to the work described.

## Authors’ contributions

This work was carried out in collaboration between all authors. Author MS designed the study and wrote the protocol and wrote the first draft. Authors MMR, AZ, MMAA and MMNU searched the literatures and collected data and wrote the first draft of the manuscript. Author HMR finalized the manuscript. All authors read and approved the final manuscript.

## Pre-publication history

The pre-publication history for this paper can be accessed here:

http://www.biomedcentral.com/1472-6874/14/54/prepub
